# Post Nasal Catarrh and Diseases of the Nose, Causing Deafness

**Published:** 1884-09

**Authors:** 


**Post Nasal Catarrh and Diseases of the Nose, Causing Deafness.** By EDWARD WOATES, M. D., Aural Surgeon and Lecturer on Diseases of the Ear, London; Senior Surgeon, Hospital for Diseases of the Throat, London. Illustrated with wood engravings. Philadelphia: P. Blakiston, Son & Co. 1884.

The volume before us deals with a department of disease subsidiary to that which is usually considered the special domain of the aural surgeon. It treats of those catarrhal diseases of the naso-pharynx of which ear diseases and deafness are secondary and later issues. The author is an able writer and a teacher of great experience, and he has embodied in the present work the results of a wide field of practical observation. This is one of the many books which have lately appeared on this subject, and we would heartily commend it. The chapters on post nasal vegetations, and on the treatment of nasal stenosis, are peculiarly good. The publishers have issued it in excellent form, and it is sufficiently illustrated.

